# Persistent postdischarge pain and chronic postoperative pain after breast cancer surgery under general anesthesia and single-shot paravertebral block: incidence, characteristics and impact on quality of life and healthcare costs

**DOI:** 10.2147/JPR.S195702

**Published:** 2019-04-16

**Authors:** Andrea Saporito, José Aguirre, Alain Borgeat, Andreas Perren, Luciano Anselmi, Roberto Poggi, Bruno Minotti, Stefano Cafarotti, Davide La Regina, Samuele Ceruti

**Affiliations:** 1 Service of Anesthesiology, Ospedale Regionale di Bellinzona e Valli, Bellinzona, Switzerland; 2 Department of Anesthesiology, Balgrist University Hospital, Zürich, Switzerland; 3 Department of Intensive Care, Ospedale Regionale di Bellinzona e Valli, Bellinzona, Switzerland; 4 Service of Anesthesiology, Ospedale Regionale di Lugano, Lugano, Switzerland; 5 Department of Emergency Medicine, St Gallen General Hospital, St Gallen, Switzerland; 6 Service of Thoracic Surgery, Ospedale Regionale di Bellinzona e Valli, Bellinzona, Switzerland; 7 Service of Visceral Surgery, Ospedale Regionale di Bellinzona e Valli, Bellinzona, Switzerland; 8 Department of Intensive Care, Geneva University Hospital (HUG), Geneva, Switzerland

**Keywords:** breast surgery, paravertebral block, regional anesthesia, postoperative pain

## Abstract

**Introduction:** Breast surgery is associated with persistent postsurgical pain; usually related to poorly treated acute pain. Paravertebral block has been successfully employed in analgesic protocols for breast surgery; its impact on postdischarge pain (PDP) has not been investigated. The aim of this study was to assess characteristics of PDP after breast surgery, the development of chronic postoperative pain (CPP) and its impact on health care costs.

**Methods:** We conducted a retrospective, observational study on a continuous cohort of adult female patients undergoing local breast cancer surgery under combined anesthesia. All patients were interviewed 6 months after hospital discharge. The survey was specifically conceived to assess incidence, features and duration of PDP. The overall cost of additional healthcare resources consumed with a specific relationship to persistent PDP was estimated.

**Results:** A database of 244 patients was preliminarily analyzed. Of these, 188 were included in the following statistical analysis; 123 patients (65.2%) reported significant PDP, with a median intensity on NRS of 6 (IQR=2), more frequently described as burning and associated with paresthesia and/or hyperalgesia (87 patients, 46%). One hundred and six patients (56.5%) reported this pain as interfering with their normal daily activities, work and sleep. In 26.8% of cases (50 patients) symptoms lasted more than 1 month and in 28 patients (15.0%) pain became chronic. The majority of patients self-treated their pain with non-steroideal anti-inflammatory drugs, but in 50 patients (26.8%) this therapy was reported as ineffective. This additional consumption of healthcare resources led to a significant economical impact.

**Conclusion:** PDP and CPP seem to be common complications after breast cancer surgery, even if a combined anesthesia technique with a thoracic paravertebral block is performed, leading to severe consequences on patients’ quality of life and increasing consumption of healthcare resources after discharge.

**Trial number: **NCT03618459 (www.clinicaltrials.gov).

## Introduction

Breast cancer is the most frequently diagnosed tumor in women, with an incidence of more than one million new cases per year.^1^ Surgery is often part of the treatment, and the prognosis has progressively improved during the last decade. There is an increasing interest in patients’ life quality after surgery, on which pain has a huge impact. In fact, breast surgery is associated with a high prevalence of chronic postoperative pain (CPP).^2^ Patients complain about persistent painful sensation in correspondence of the surgical wound, lasting beyond 2 months since the surgical intervention. This has progressively emerged as a relatively frequent delayed complication of breast surgery, being its prevalence of up to 60%.^2^ Moreover, this disabling condition significantly affects cancer survivors’ quality of life^3^,^4^ and to have an impact on healthcare costs.^5^,^6^

Development of chronic postbreast surgery pain is associated with some potential risk factors.^2^ Of all the predictors of CPP, which have been currently identified in the literature as local pain persisting more than 2 months after surgery,^7^ a recurring risk factor seems to be poorly treated acute pain.^7^,^8^

Recently, the awareness of the social impact of untreated postoperative pain is broadening the debate regarding the translation from chronic wound pain to persistent postoperative pain;^7^ the latter, even if not meeting the criteria to be classified as chronic, has been recognized as a major cause of unexpected hospital readmission and a detrimental factor affecting patients’ quality of life.^7^,^9^

A Cochrane systematic review has addressed the role of regional anesthesia in preventing the development of CPP, suggesting that “paravertebral block may reduce the pain after breast cancer surgery in about one out of five women treated“, these results being however weakened by a poor quality or an inadequate power of the studies included.^10^,^11^ The mechanism underlying of regional anesthesia could be a preventive effect on the remodeling of the nervous system, which occurs when a persistent nociceptive stimulus is applied and often results in hyperalgesia, allodynia and a sustained wound pain.^11^

Even though CPP has been progressively recognized as a significant clinical issue after breast cancer surgery,^12^ the link between the anesthetic regimen and the postdischarge pain (PDP) has been poorly investigated. Single-shot paravertebral block is an effective technique to provide postoperative analgesia in the first postoperative hours after breast surgery, especially in patients who are not undergoing axillary surgery, but its long-term benefits are still debated.^8^ The aim of this study is to assess incidence, characteristics and both clinical and economic consequences of PDP, as well as its correlation with the incidence of CPP development in a continuous cohort of patients undergoing local breast cancer surgery in a standardized combined anesthesia regimen, including a single-shot analgesic paravertebral block.

## Material and methods

We performed a retrospective observational study on a consecutive cohort of female adult patients who underwent local breast cancer surgery with a standardized combined anesthesia technique, including a standardized single-shot thoracic paravertebral block performed before surgery with a long-lasting local anesthetic and general anesthesia, which is described in more detail in the section below.

As part of the local quality survey aimed at assessing patients’ satisfaction with specific regard to the quality of postoperative analgesia after breast surgery, all patients undergoing breast cancer surgery were systematically telephone interviewed 6 months after hospital discharge. The survey, conducted after the inclusion of paravertebral block in the postoperative analgesia protocol for breast cancer surgery, was specifically aimed at assessing, besides overall satisfaction, the incidence, features and duration of an eventual PDP. Answers to the questionnaire were anonymously registered in an electronic database.

After approval by the Institutional Review Board of the Hospital (Bellinzona Regional Hospital, Bellinzona, Switzerland)Ospedale Regionale di Bellinzona e Valli, an observational retrospective cohort study was conducted on the results of the survey. The study was registered on Clinicaltrial.gov (number NCT03618459) and was carried out according to the principles of the Declaration of Helsinki. Inclusion criteria for the analytic cohort were: 1) consecutive ASA 1–3 adult female patients, 2) elective local breast surgery, namely unilateral tumor resections, lumpectomies or mastectomies, without axillary lymphadenectomy, 3) under a combined anesthesia with a standardized single-shot thoracic paravertebral block and 4) who completed the telephone interview 6 months after surgery. Patients were excluded if any data at any time regarding their pain evolution and medications were incomplete.

### Combined anesthesia

All patients underwent the same standardized anesthetic treatment, according to a specific protocol. After arrival in the preoperative induction room, standard monitoring and peripheral venous access were attained. A standard procedural intravenous analgesia and sedation was administered, with 1 μg/kg of fentanyl and 1–2 mg of midazolam. All patients underwent an ultrasound-guided unilateral thoracic paravertebral block both at T_H3_ and T_H4_ level using an in-plane approach with a 5 cm needle (Contiplex D, B. Braun Medical Inc., Melsungen, Germany), according to a standardized procedure. After a preliminary orientation scan aimed at identifying the correct levels and prior to the procedure, skin disinfection was performed with a two-layer application of an alcoholic povidone-iodine solution (Betaseptic®, Mundipharma, Basel, Switzerland). Three minutes later, the area of the puncture point was covered with sterile drapes. After confirmation of correct positioning of the needle tip with the administration of a small bolus of normal saline under ultrasound guidance and after negative aspiration, two boluses of 10 mL of ropivacaine 0.75% (Naropine, AstraZeneca plc, London, UK) were administered both at T_H3_ and T_H4_, for a total volume of 20 mL, under real-time ultrasound visualization. Block success was subsequently evaluated with thermal sensation testing: a block was defined as successful when associated with a complete loss of cold perception overall dermatomes involved in the planned surgery 20 mins after local anesthetic injection.

Thereafter, total intravenous anesthesia was induced after further administration of 1–2 mcg/kg of fentanyl prior to laryngeal mask placement. During general anesthesia, all patients received a single dose of diclofenac 75 mg and paracetamol 1 g intravenously as per local protocol. Systemic postoperative analgesia with oral diclofenac (50 mg tid) and paracetamol (1 g qd) was always prescribed during the whole hospital stay.

### Questionnaire

Data were collected with a specific questionnaire, including information such as the date of surgery, type and site of surgery, and basal patients’ demographic data. Patients were subsequently contacted by phone at home, 6 months after surgery, concerning their pain’s evaluation and the adequacy of own postoperative analgesia during the first 24 hrs after surgery, stratifying it into categorical variables as insufficient*,* sufficient, good or perfect. The analgesic protocol was considered as effective when patients evaluated their analgesia as sufficient, good or perfect.

Patients were subsequently asked to discuss the occurrence of PDP. PDP was evaluated by Numerical Rating Scale (NRS, from 0 intended as no pain, to 10 as maximal pain), defining “pain” any ache referred as “more than 2”. Data regarding PDP duration and its characteristics (burning versus stubbing, associated with paresthesia or to any neurologic symptom), localization (local and/or regional and relative distribution), impact on daily life activity (particularly on work, leisure time or sleep) and treatment (classified according to use of paracetamol and/or non-steroideal anti-inflammatory drugs (NSAID) and/or oral opioids) were registered.

### Outcome measures

The primary outcome of the study was the incidence of PDP. Secondary outcomes were: PDP characteristics, impact on patient daily life after discharge, and the correlation between PDP and the incidence of CPP, defined as wound pain a period of 3 months after surgery. Another secondary outcome was the postoperative healthcare costs associated with analgesic drugs consumption and consultations with the general practitioners related to pain issues.

### Statistics

Unpaired Student's *t*-test was used for parametric data null hypothesis testing, while Mann–Whitney *U*-test was applied to non-parametric data. A *P*-value<0.05 was considered statistically significant (95% CI). Results are given as mean (m) ± SD for normally distributed parameters or median (M) and relative IQR for non-normally distributed parameters. A Chi-square test was performed in order to evaluate any statistical correlation between PDP and CPP. The economic analysis was performed assuming the point of view of the community. Postdischarge healthcare costs were evaluated with a top-down technique by computing the costs of consultations by general practitioners (based on recall from patients) and analgesic drugs. The costs of lost working days due to pain-related disabilities were not taken into account. Costs were expressed in USD. Statistical analysis was performed using SPSS (IBM Corporation, Armonk, NY, USA) and Numbers ’09 2.1 version (Apple Inc., Bellinzona, Switzerland) software. All data were collected and stored anonymously in an electronic protected database.

## Results

Of 244 consecutive questionnaires, 56 were excluded because incomplete, while 188 were considered in the final analysis. The main characteristics of the population were: mean age 58.9±14.6 years, median ASA class 2 (IQR=1) and mean length of hospital stay 6±1 days.

After discharge at home, 70.2% (N=132) of patients referred localized (uniquely at the site of the surgical wound) or regional PDP radiating to the ipsilateral arm (Table 1). Median PDP intensity on NRS was 6 (IQR=2), and pain was more frequently described as burning and associated with paresthesia and/or hyperalgesia (46%), rather than stabbing, suggesting a neuropathic component. In a significant proportion of patients (14.9%) PDP had a negative impact on many aspects of their lives, such as sleep, recreational activities and work abilities, but patients were hardly able to point out only one of these aspects as predominant over others.

**Table 1:** Primary outcome and main secondary outcomes

Primary outcome		
PDP incidence, no. (%)	132 (70.2%)	
**Secondaries outcomes**		
PDP intensity, median (IQR)	6 (2)	
PDP characteristic	Burning with paresthesia/hyperalgesia (46%)	
Disability incidence, no. (%)	28 (14.9%)	
Pain 24 hrs after surgery	Perfect	49%
	Good	38%
Sufficient	10%
	Insufficient	3%
Pain treatment during whole global stay	Perfect	37%
	Good	55%
Sufficient	5%
	Insufficient	3%
** **PDP, no. (%)	More than 1 month	50 (26.6%)
More than 3 months	28 (14.9%)
	At 6 months	16 (8.5%)
Pharmacologic treatment, no. (%)	Self treated	123 (65.2%)
	Treated but not efficacy	50 (26.7%)
Treated and need a visit	35 (18.8%)

**Notes:** For details, please refer to the text. “Disability incidence” is defined as severe impairment of daily activities.

**Abbreviation:** PDP, postdischarge pain.

### In-hospital pain treatment

**Figure 1:**
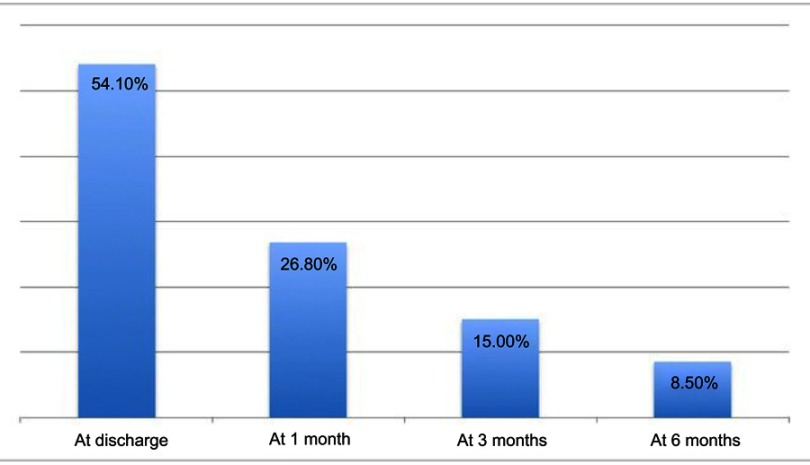
Percentage of patients affected by postdischarge pain at discharge, at 1 month, at 3 months and during the interview at 6 months. Median Numerical Rating Scale pain perception was 6 (IQR=2).

**Figure 2:**
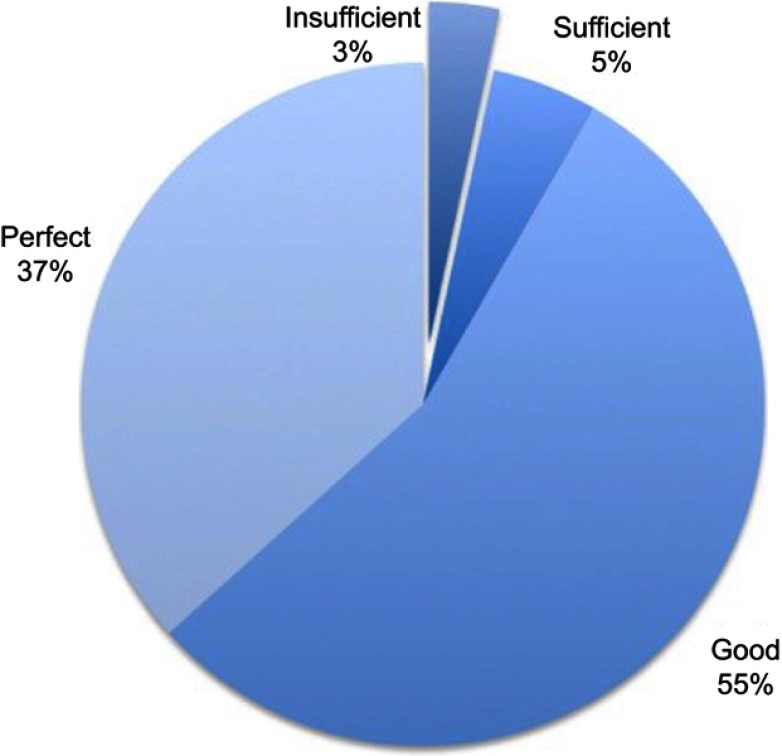
Patients’ evaluation of regional anesthesia effect. Regional anesthesia was considered adequate if evaluated as sufficient, good or perfect.

Regarding efficacy of acute pain treatment the day after surgery, patients evaluated their analgesia at awakening from general anesthesia as perfect in 49%, good in 38%, sufficient in 10% and inadequate in 3% of cases (Figure 1). Acute pain management was thus considered adequate in about 97% of patients. Regarding patients’ analgesia over the whole hospitalization assessed during phone recall, patients rated it as perfect in 37% of cases, good in 55% of cases, sufficient in 5% of cases and inadequate in 3% of patients (Figure 2). Overall inpatient analgesia was then considered adequate in 97% of patients.

### PDP and chronic pain

**Figure 3:**
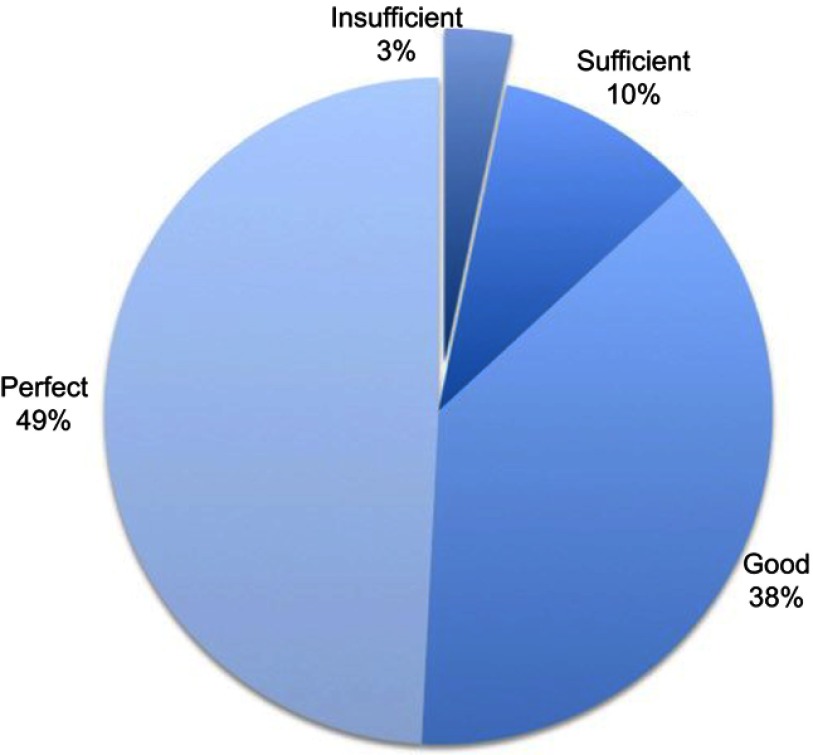
Global intra-hospital analgesia efficacy, evaluated by patients. Global analgesia was considered adequate if evaluated as sufficient, good or perfect.

In 26.6% of patients (n=50), PDP persisted for more than 1 month and in 14.9% (n=28) pain became chronic, longer than 3 months duration; in 8.5% of these (n=16), PDP was still present during the interview (Figure 3). Good in-hospital analgesia did not seem to prevent the development of PDP: most of the patients considered their in-hospital analgesia as at least sufficient.

A high correlation was found between PDP and the development of CPP: all patients (188 patients, 100%) developing a CPP referred to having suffered from PDP immediately after home discharge. No patient who was PDP-free after discharge subsequently developed a CPP (*p*<0.001).

### PDP and healthcare costs

The majority of patients affected by PDP (123 patients, 65.2%) treated their pain with a combination of paracetamol and NSAIDs – the most common was diclofenac; in 26.7% of cases (50 patients) this therapy was ineffective and 18.8% of patients went to their general practitioner for a consultation related to their PDP. During the first 6 months postoperatively, postdischarge costs of home-based systemic analgesia and eventual general practitioner consultations related to pain issues were significantly higher in patients with PDP compared to patients without PDP, with an estimated increase in healthcare related costs per patient over the first 6 postdischarge months of 1,367 USD, due to medications (about 900 USD for 6 months) and consultation with general practitioners specifically attributable to PDP or PDP-related issues (about 400 USD during 6 months).

## Discussion

The preventive role of regional anesthesia on CPP in relation to its effective control of acute pain has often been postulated. Hussain et al,^11^ have shown that a combined anesthesia regimen including a paravertebral block to be associated with a better acute postoperative analgesia compared to controls. However, regional anesthesia does not exclude the possibility to develop a persistent wound pain. Little is known about the characteristics and the duration of PDP in this particular population, undergoing breast cancer surgery with a paravertebral block, which was the aim of this study. In this study, both PDP and CPP after breast surgery have been shown to be common, also when a combined anesthesia technique is adopted, with a single-shot thoracic paravertebral block performed before induction. In particular, PDP was referred by the majority of the patients interviewed and had an important negative impact on their quality of life, affecting both normal working activity and sleeping in a very high percentage of cases. Pain incidence in this study is actually quite low compared to prior studies on this topic;^11^ this is likely due to the employed regional anesthesia, but it is possible that the incidence of pain may be less frequently depending on differences about demographic data (like age), postoperative acute treatment and pain definition.

A single shot paravertebral block seems to be a good choice to provide effective in-hospital analgesia during the first days after surgery, but does not seem to prevent the development of PDP in the following period.

PDP duration was extremely variable, often lasting for many weeks after surgery and evolving into chronic pain syndrome in 15% of cases. Patients generally attempted to self-medicate PDP with NSAIDs, treatment turning out to be ineffective and leading eventually to a visit to the family practitioner, with an obvious impact on patient’s life quality and total healthcare costs.

Previous studies focused on CPP incidence, while this is the first study specifically addressing PDP after local breast surgery, with the aim of quantifying and characterizing this condition, its dimension and gravity of which was found to be a potentially important healthcare issue. Our finding supports the hypothesis that, even when a good anesthetic plan is routinely adopted and includes regional anesthesia technique such as a single shot-paravertebral block with a long-lasting local anesthetic, PDP is not prevented. Moreover, CPP whose incidence is still high in this specific group of patients is also not prevented by a single-shot paravertebral block.

PDP in breast surgery patients seemed to have specific neuropathic features, which is a significant subgroup of patients interviewed had an important and prolonged impact on quality of life and possibly on postdischarge healthcare costs, ultimately causing a sustained use of analgesic drugs and consultations by general practitioners for pain-related issues. It is important to notice that our patients were not enrolled in a fast-track program and stayed in the hospital for several days before discharge home. During their hospitalization, they received systemic analgesia with NSAID, paracetamol and rescue opioids on demand, according to a standard protocol. However, even if patients remained in hospital and were monitored for days, being regularly treated with analgesic drugs, PDP and the development of CPP were not prevented.

Chronic pain is well known to be a public health issue: 22% of US primary care appointments focus on pain management, and a linear trend of specific national pain consultations has been reported,^7^,^13^ showing an increase from 11% to 14% over the period 2000–2007 in the US. Moreover, patients suffering from chronic pain resulted in seeing their general practitioners up to five times more frequently than other patients.^13^,^14^ Total costs of prescribed pain medication are $17.8 billion annually, only in the US, and the largest three categories for pain therapy costs were $1.9 billion for analgesics/NSAIDs, $3.6 billion for opioids and $12.3 billion for adjuvants.^15^,^16^ Unfortunately, there are no Swiss data available on this specific topic, despite that drugs consumptions in Switzerland are known to be the biggest in Europe and second only to US.^17^

Further studies are required to define effective strategies to treat postoperative pain after breast surgery specifically aiming at preventing PDP, eventually decreasing the incidence of postoperative chronic pain. Regional anesthesia as well as the preventive use of systemic drugs, such as ketamine, have been shown by some preliminary evidence to be possibly associated with a reduction in chronic pain incidence;^18^ but these data must still be confirmed by adequately designed and powered studies, particularly in the context of breast surgery.

Our study has several limitations. Firstly, this was a single-center retrospective study, including all relative drawbacks compared to prospective randomized trials. Secondly, only one analgesic management was studied, making results applicable only to this therapeutic management, even if the paravertebral block is considered as an advanced technique to control acute pain after breast surgery. Moreover, our study does not stratify patients with regard to eventual postoperative treatments which may potentially influence the intensity, the characteristics and the duration of postoperative pain, such as radiotherapy.

For the assessment of the characteristics of PDP, we did not use a validated scale (like VAS-scale), as we wanted to focus on a combination of factors, assessing the duration, the intensity and the characteristics of pain. Another limitation is that, in order to adequately assess PDP and CPP incidence, our questionnaire was performed 6 months after hospital discharge, which might have affected patients’ accuracy in assessing their pain. However, this may also represent an advantage with regard to the possibility of giving a more detached and unbiased evaluation of their entire postoperative experience overall.

## Conclusion

PDP and CPP seem to be common complications after breast surgery even when a general anesthesia technique is combined with a single-shot thoracic paravertebral block. Given the impact of these complications on the patients’ quality of life and healthcare costs, alternative analgesic strategies and a proper follow-up seem to be recommendable, in order to prevent or early detect and adequately treat PDP.

## Data sharing statement

All dataset used and/or analyzed during the current study is available from the first author on reasonable request made by email for a period of 10 years from the closing of the study.
